# Physiological responses and performance factors for double-poling and diagonal-stride treadmill roller-skiing time-trial exercise

**DOI:** 10.1007/s00421-023-05239-8

**Published:** 2023-06-11

**Authors:** Erik P. Andersson, Nestor Lögdal, Darragh Byrne, Thomas W. Jones

**Affiliations:** 1 Swedish Winter Sports Research Centre, Department of Health Sciences, Mid Sweden University, Östersund, Sweden; 2 School of Sport Sciences, Faculty of Health Sciences, UiT the Arctic University of Norway, Tromsø, Norway; 3 Centre for Musculoskeletal Research, Department of Occupational and Public Health Sciences, University of Gävle, Gävle, Sweden; 4 Department of Sport, Exercise and Rehabilitation, Northumbria University, Newcastle upon Tyne, UK

**Keywords:** Anaerobic capacity, Cross-country skiing, Gross efficiency, Pacing strategy, Peak oxygen uptake

## Abstract

**Purpose:**

To compare physiological responses between a self-paced 4-min double-poling (DP) time-trial (TT_DP_) versus a 4-min diagonal-stride (DS) time-trial (TT_DS_). The relative importance of peak oxygen uptake (${\dot{\text{V}}}$O_2peak_), anaerobic capacity, and gross efficiency (GE) for projection of 4-min TT_DP_ and TT_DS_ roller-skiing performances were also examined.

**Methods:**

Sixteen highly trained male cross-country skiers performed, in each sub-technique on separate occasions, an 8 × 4-min incremental submaximal protocol, to assess individual metabolic rate (MR) versus power output (PO) relationships, followed by a 10-min passive break and then the TT_DP_ or TT_DS_, with a randomized order between sub-techniques.

**Results:**

In comparison to TT_DS_, the TT_DP_ resulted in 10 ± 7% lower total MR, 5 ± 4% lower aerobic MR, 30 ± 37% lower anaerobic MR, and 4.7 ± 1.2 percentage points lower GE, which resulted in a 32 ± 4% lower PO (all *P* < 0.01). The ${\dot{\text{V}}}$O_2peak_ and anaerobic capacity were 4 ± 4% and 30 ± 37% lower, respectively, in DP than DS (both *P* < 0.01). The PO for the two time-trial (TT) performances were not significantly correlated (*R*^2^ = 0.044). Similar parabolic pacing strategies were used during both TTs. Multivariate data analysis projected TT performance using ${\dot{\text{V}}}$O_2peak_, anaerobic capacity, and GE (TT_DP_, *R*^2^ = 0.974; TT_DS_, *R*^2^ = 0.848). The variable influence on projection values for ${\dot{\text{V}}}$O_2peak_, anaerobic capacity, and GE were for TT_DP_, 1.12 ± 0.60, 1.01 ± 0.72, and 0.83 ± 0.38, respectively, and TT_DS_, 1.22 ± 0.35, 0.93 ± 0.44, and 0.75 ± 0.19, respectively.

**Conclusions:**

The results show that a cross-country skier’s “metabolic profile” and performance capability are highly sub-technique specific and that 4-min TT performance is differentiated by physiological factors, such as ${\dot{\text{V}}}$O_2peak_, anaerobic capacity, and GE.

## Introduction

In traditional classic-style cross-country skiing races over hilly terrain, diagonal stride (DS) and double poling (DP) are the two most frequently employed sub-techniques (Sandbakk et al. 2016b). Typically, DS is employed on uphill sections at speeds that range from ~ 1.5 to ~ 4.5 m·s^−1^, whereas DP is employed on flatter course sections, and slight downhill sections, at speeds that range from ~ 4.0 to ~ 9.0 m·s^−1^ (Losnegard 2019). In DS, both arms and legs are involved in the active propulsion with diagonal arm and leg movements (similar to running and walking), whereas DP solely involves active propulsion via the arm-poling action without any active propulsion from the legs. Today, long-distance cross-country ski races that are included in the long-distance ski championship are performed on flatter courses than the traditional world-cup races and are mainly won by skiers exclusively using DP. To prevent the traditional sub-techniques from becoming nonexistent, the International Ski Federation (FIS) recently introduced technical zones on certain uphill sections of competition tracks where DP is forbidden (Stöggl et al. 2019), which in combination with hilly course profiles will mean that DS continues to be an important sub-technique in the traditional races of cross-country skiing.

Irrespective of exercise modality, it has been demonstrated that whole-body peak oxygen uptake (${\dot{\text{V}}}$O_2peak_), or maximal oxygen uptake (${\dot{\text{V}}}$O_2max_), increases when the arms contribute 10–30% of the total power output, while ${\dot{\text{V}}}$O_2peak_ decreases when the arms contribute > 30% of the total power output (Bergh et al. 1976). When comparing the DP and DS sub-techniques of cross-country skiing, ${\dot{\text{V}}}$O_2peak_ in DP, which involves a high level of arm activity, is ~ 12% lower than in DS (Losnegard 2019). The lower ${\dot{\text{V}}}$O_2peak_ in DP than in DS has from a physiological perspective been related to a lower oxygen extraction as well as a lower cardiac output mainly driven by a lower peak heart rate (Andersson et al. 2021; Björklund et al. 2015; Calbet et al. 2005; Stöggl et al. 2013). An additional important performance factor in cross-country skiing is the anaerobic energy supply (Gløersen et al. 2020; Losnegard et al. 2012). Anaerobic capacity has been shown to account for a large portion of the variation in performance, both between (Losnegard et al. 2012) and within athletes, during repeated roller-skiing sprint time trials (Andersson et al. 2016) and has also shown to be important for uphill-section performance during distance races (Gløersen et al. 2020). In addition, the ability to recover the anaerobic energy system is highly important during distance races over undulating terrain (Gløersen et al. 2020).

In an endurance sport such as cross-country skiing, external power output is from a solely physiological perspective dependent on the sum of aerobic and anaerobic metabolic rates (in W) multiplied by gross efficiency (GE) (Andersson et al. 2017). Due to the differing speeds and muscular contributions between DP and DS, it is unsurprising that physiological variables, such as ${\dot{\text{V}}}$O_2peak_, anaerobic capacity, and GE, differ between the two sub-techniques, which is a unique aspect of cross-country skiing (Andersson et al. 2017). Data indicate that both ${\dot{\text{V}}}$O_2peak_ and anaerobic capacity are lower for “flat” DP (1–2° incline) than uphill DS (7° incline) cross-country skiing, which together with the considerably lower GE (~ 3–4 percentage points) would result in a substantial difference in external power output between the two sub-techniques (Andersson et al. 2017; Sandbakk et al. 2016a). Moreover, recent research has shown a higher between-athlete variation in GE for DP than DS (Andersson et al. 2017). In addition, the relationship between GE and speed (or power output) has been observed to be speed dependent in DP, but not in DS (Andersson et al. 2017), probably due to differences in cycle characteristics and force application patterns as well as the different muscle recruitment and muscle contraction properties between the two sub-techniques (Losnegard 2019).

From a physiological perspective, high-level endurance athletes seem to be relatively heterogeneous in their respective physiological strengths and weaknesses as indicated by the relatively low strength of separate pair-wise correlations between physiological performance factors and performance (Andersson et al. 2017; Laaksonen et al. 2020). Since the finish time in a traditional classic cross-country skiing race on a standard FIS course is related to the sum of all sub-technique-specific performances, the best race performances are characterized by a high DP and DS-specific performance (Sandbakk et al. 2016b). However, the locomotion of DP and DS differ substantially (Pellegrini et al. 2013) and upper body-specific physiological characteristics are likely to be more crucial for DP than DS performance (Stöggl et al. 2019). Due to this, a skier that performs well in DS may not necessarily perform well in DP, or vice versa, and physiological characteristics in one sub-technique, such as ${\dot{\text{V}}}$O_2peak_, anaerobic capacity, and GE may not directly transfer to the other sub-technique.

Although a traditional incremental ${\dot{\text{V}}}$O_2max_ test may be an adequate test for the assessment of ${\dot{\text{V}}}$O_2max_, it is not a reliable and/or race-specific type of test (Jeukendrup et al. 1996; Noakes 2008). In comparison to a traditional incremental ${\dot{\text{V}}}$O_2max _test, a short time-trial test (∼4 min) may be a preferable alternative as a laboratory-based performance test as it is more reliable and can be used for the assessment of ${\dot{\text{V}}}$O_2max_ and anaerobic capacity (McGawley 2017; Watkins et al. 2017). In addition, GE can be determined during the submaximal warm-up exercise (Andersson and McGawley 2018).

Recent research reveals a relatively large between-athlete variation in sub-technique-specific performance differences between DP and DS (Andersson et al. 2017, 2021; Sagelv et al. 2018; Stöggl et al. 2019), which requires further investigation. Research shows that it seems difficult to increase the ${\dot{\text{V}}}$O_2max_ that is reached during whole-body exercise (usually uphill DS) in already highly trained senior-elite cross-country skiers (Losnegard et al. 2013). Therefore, reducing sub-technique-specific differences in physiological capabilities between DP and DS and/or focusing on improving GE in “weaker” sub-techniques seems like an appropriate training strategy for further performance enhancement in already highly trained athletes. To date, there is a lack of information regarding differences in performance and physiological responses between DP and DS in highly trained senior male cross-country skiers based on a duration-specific time-trial performance test. Therefore, the primary aim of the current study was to compare physiological and perceptual responses between a self-paced 4-min DP time-trial (TT_DP_) performance versus a 4-min DS time-trial (TT_DS_) performance. A secondary aim was to examine the relative importance of ${\dot{\text{V}}}$O_2peak_, anaerobic capacity, and GE for the projection of 4-min TT_DP_ and TT_DS_ performances.

## Materials and methods

### Participants

Sixteen highly trained male cross-country skiers (26 ± 5 years, 182 ± 6 cm, 77.3 ± 6.7 kg), competing at a national level and/or an international level (Tier 3, *n* = 10; Tier 4, *n* = 6 [according to McKay et al. (2022)], were recruited for the study that was performed ~ 2–3 weeks after their race season. The participants' distance and sprint FIS points were 70 ± 23 and 135 ± 43, respectively (for details about FIS points, see Jones et al. (2021)). Of the 16 participants, three did not compete in sprint races and had due to this no FIS points. Participants were instructed to engage only in low-intensity exercise (1-h maximum) the day prior to testing and consume carbohydrate-rich meals. The study was preapproved by the Regional Ethical Review Board of Umeå University, Umeå, Sweden (#2018–154-31 M). Participants received both written and verbal information about the experimental protocol and possible risks involved, before providing written informed consent.

### Study overview

On separate test days, participants completed in each sub-technique (DP and DS) a continuous incremental submaximal protocol consisting of eight 4-min bouts at intensities between ~ 47–78% of ${\dot{\text{V}}}$O_2peak_ that was followed by a short break (10 min) and thereafter a self-paced 4-min roller-skiing TT performance test (i.e., TT_DP_ or TT_DS_) at maximal effort all performed on a treadmill with automated speed control. The two test days were completed within 2 weeks, separated by at least 2 days, and the order of sub-technique was randomized. The inclination of the treadmill was set to 1.5° and 6.5° for the DP and DS roller-skiing tests, respectively, as these are the typical gradients (on average) where the DP and DS sub-techniques are used (for details, see Losnegard (2019)). All participants were familiarized with the specific tests and the testing procedure.

### Equipment and measurements

All tests were performed on a treadmill specifically designed for roller-skiing (Rodby Innovation AB, Vänge, Sweden) that allows the athlete to freely adjust the speed and distance completed during the TT was automatically logged (2.46 Hz) and linearly interpolated to second-by-second data. Participants completed all testing using the same pair of classical roller skis (Pro-Ski C2, Sterners, Dala-Järna, Sweden) with the coefficient of rolling resistance (μR) being 0.0215 and determined according to Ainegren et al. (2008). To avoid changes in rolling resistance during test sessions, roller-skis were pre-warmed in a heat box for a minimum of 60 min prior to testing. Participants used their own poles, which were fitted with custom-made rubber tips designed for treadmill skiing, and the same pair of poles was used for both the DP and DS tests. Respiratory measurements were performed using an AMIS 2001, model C (Innovision AS, Odense Denmark). The gas analyzers were calibrated with a known reference gas mixture (16.0% O_2_ and 4.5% CO_2_, Air Liquide, Kungsängen, Sweden) and ambient air. The flowmeter was calibrated with a 3-L syringe at low, medium, and high flow rates (Hans Rudolph, Kansas City, Missouri, USA). Calibration was performed before the start of each test. The ambient temperature was 19.5 ± 0.5 °C at a relative humidity of 21 ± 6% which was monitored with a Vaisala PTU200 (Vaisala Oy, Helsinki, Finland). A Biosen S_Line (EKF diagnostics, Magdeburg, Germany) equipment was used to determine the blood lactate concentration, which was calibrated with a known standard solution of 12 mmol·L^−1^.

All equipment used for the roller-ski assessments was validated prior to the test period. Treadmill speed and incline were validated using an electronic tachometer (Lutron Electronic Enterprise CO, Taipei, Taiwan) and a digital inclinometer (DNM 60 L Pro, Bosch GmbH, Germany), respectively. The ergospirometry AMIS system was validated against a mechanical lung simulator (Metabolic Simulator No 17056, Vacumed, Ventura, CA, USA) and custom-made Douglas bags. Relative concentrations and volumes of expired gas were analyzed using a MOXUS Metabolic Cart (AEI technologies, Bastrop, TX, USA) and a custom-built spirometer (Fabri AB, Spånga, Sweden). The AMIS system was also validated across a wide range of submaximal workloads corresponding to oxygen uptakes between 0.7 and 5.0 L·min^−1^ and a respiratory exchange ratio < 1.00. The typical error in ${\dot{\text{V}}}$O_2_ values prior to testing was < 0.1 L·min^−1^.

### Testing procedures

Upon arrival at the laboratory body mass of the participants, with and without equipment was measured using an electronic scale (Seca 764, Hamburg, Germany) followed by a 5-min supine rest. The DP protocol was performed at an incline of 1.5° and the DS protocol was performed at an incline of 6.5°. The starting speed was either 6 or 6.5 km·h^−1^ for the DS protocol and either 12.6 or 13.8 km·h^−1^ for the DP protocol based on previous race results and/or the familiarization. The speed was increased by 0.5 km·h^−1^ up to a final speed of either 9.5 or 10 km·h^−1^ for DS, whereas the speed was increased by 1.2 km·h^−1^ up to a final speed of either 21 or 22.2 km·h^−1^ for DP. Both protocols consisted of 8 × 4-min submaximal stages (except for the first stage which lasted 8 min), followed by a 10-min passive rest and a 4-min self-paced TT at a maximal effort. The participants were instructed to cover as much distance as possible during the self-paced TT. Participants received feedback on elapsed time every 30 s but received no feedback regarding their speed during the TT. Participants completed a familiarization session on the treadmill before their first test day to minimize the effect of learning on time-trial (TT) performance. This involved submaximal skiing with DP (3 × 5 min) and DS (3 × 5 min) at fixed speeds of 13, 17, and 21 km·h^−1^ for DP and 6.0, 7.5, and 9.0 km·h^−1^ for DS and 10 min of varied intensity skiing (5 min each for DP and DS) using the automated speed control system. This was followed by a short break and a race-paced 4 min TT_DP_/TT_DS_ in a randomized order, with approximately 20 min of recovery between the TTs.

A capillary blood sample (20 μL) was taken from the fingertip for the assessment of blood lactate concentration 2 min after the TT. The skiers rated their perceived exertion (RPE) after the last submaximal stage as well as immediately after the TT using the 10-point scale of Foster et al. (2001) and retrospectively at minutes 1, 2, and 3 of the TT. During the submaximal protocol and TT, both respiratory variables and heart rate were collected continuously. The highest 30-s moving average during the TT was used to calculate ${\dot{\text{V}}}$O_2peak_ and peak ventilation rate. Peak respiratory exchange ratio (RER) was taken over the same period as ${\dot{\text{V}}}$O_2peak_. Peak oxygen pulse was calculated as ${\dot{\text{V}}}$O_2peak_ divided by heart rate (30-s average) at ${\dot{\text{V}}}$O_2peak_. Participants were secured with a safety harness suspended from the ceiling and connected to an emergency brake during all testing that stopped the treadmill in case of a fall.

### Calculations

#### Power output, gross efficiency, and metabolic responses

The power output for submaximal roller-skiing at constant speed was calculated as the sum of the power exerted to overcome the rolling resistance and to elevate system mass (SM) (i.e., body mass and skiing equipment) against gravity

1 $$\mathrm{Power \,output} \left[W\right]= vSM\left(g \,\mathrm{sin}\left(\alpha \right)+{\upmu }_{R}g\, \mathrm{cos}\left(\alpha \right)\right),$$

where *g* is gravitational acceleration,* v* is the treadmill speed [m·s^−1^], µ_*R*_ is the rolling resistance coefficient, and *α* is the treadmill incline. Gross energy expenditure was calculated from oxygen uptake (${\dot{\text{V}}}$O_2_ [L·min^−1^]) and RER (${\dot{\text{V}}}$CO_2_· ${\dot{\text{V}}}$O_2_^−1^) according to the equation introduced by Weir (1949) and then converted into a metabolic rate

2 $$\mathrm{Metabolic\, rate} \left[W\right]= \frac{4184{(\dot{\mathrm{V}}\mathrm{O}}_{2}\left(1.1\mathrm{RER}+3.9\right))}{60}.$$

The GE was calculated using the following equation:

3 $$\mathrm{GE}= \frac{\mathrm{Power\, output} (W)}{\mathrm{Metabolic\, rate} (W)},$$

where metabolic rate was based on the average ${\dot{\text{V}}}$O_2_ and RER values (≤ 1.00) during the final minute of each submaximal bout.

For determining the most appropriate method used for calculating the anaerobic metabolic rate and anaerobic capacity in DP and DS, a previous methodological study (Andersson et al. 2020) has been published on parts of the data that are included in the current study. Based on the results presented by Andersson et al. (2020), a second-degree polynomial regression model would be more appropriate for DP roller-skiing, while a linear model would be more appropriate for DS roller-skiing; both without using a baseline metabolic rate as a Y-intercept.

For DS, a linear relationship between treadmill power output and metabolic rate during the final minute of each of the 8 × 4-min submaximal stages was derived and used to estimate the instantaneous required metabolic rate during the 4-min TT (MR_TT_req_) at each 1-s time-point. The same procedure was used for DP, with the exception that a second-degree polynomial regression was used. The power output during the TT (PO_TT_) was calculated according to Eq. 1.

The instantaneous anaerobic metabolic rate (MR_AN_) at each 1-s time-point (*t*) of the TT was expressed as

4 $${\mathrm{MR}}_{AN,t} \left[{J\cdot s}^{-1}\right]= {\mathrm{MR}}_{TT\_req,t}- {\mathrm{MR}}_{\mathrm{AE},t},$$

where MR_AE_ is the aerobic metabolic rate calculated as described in Eq. 2.

The total anaerobic energy production (in joules) was calculated by integrating MR_AN_ over the 4-min TT.

To be able to compare the average estimated supramaximal GE during the TT based on the regression equation, the following calculations were performed. First, the estimated instantaneous GE at each 1-s time-point (*t*) of the 4-min TT was calculated as the ratio between PO_TT_ (calculated similarly as in Eq. 1) and the MR_TT_req_ derived from the polynomial regression equation in DP and the linear regression equation in DS. Second, the estimated instantaneous GE was expressed as an average value in DP and DS, respectively. The instantaneous GE was also used to calculate the time-course of aerobically attributable power output (i.e., the aerobic contribution to power output) and was calculated as the instantaneous MR_AE_ multiplied by instantaneous GE.

#### Cycle characteristics and poling power

For all kinematical analyses, all tests were filmed from the side with a Go-Pro camera (GoPro Hero 1, GoPro Inc., San Mateo, CA, USA). A skiing cycle was defined as the moment from the start of the pole plant (i.e., first pole-belt contact) until the same pole made contact again with the treadmill belt. The number of complete cycles within the last minute of each submaximal stage was counted and the exact times were noticed. For the calculation of cycle rate (in Hz, i.e., cycles·s^−1^), the total number of cycles was divided by the exact time (in s) taken to complete those cycles, whereas cycle length (in m) was calculated by dividing speed (in m·s^−1^) by cycle rate. Cycle rate and cycle length were determined for both DP and DS. For DP, the times of active propulsion (i.e., the poling time) and no propulsion (i.e., the swing time) were determined based on the last five completed cycles within the final minute of each submaximal stage. Poling time was then determined as the pole-belt contact time and the swing time as the time of no pole-belt contact. Poling and swing times were presented as the average value of those five cycles. To allow for the computation of relative poling and swing times in DP (presented as percentages of cycle time), the average cycle time was calculated for the same five cycles. The average power output during the propulsive poling phase (i.e., poling power) in DP was determined as the power output (Eq. 1) divided by the poling to cycle time ratio (i.e., the relative poling time).

Average values for cycle rate and cycle length were calculated for each 1-min period of the TT_DP_ and TT_DS_. For the calculation of cycle rate, the number of full cycles that were completed within each respective minute was counted and cycle rate was calculated as the number of completed cycles divided by the exact time. Cycle length was calculated as the average speed for the specific period divided by cycle rate.

### Statistics

Data were checked for normality by visual inspection of Q–Q plots and histograms together with the Shapiro–Wilk analysis and are presented as mean ± standard deviation (SD), except in the case of RPE, where data are presented as median and interquartile range (IQR). One-way repeated-measures ANOVA tests were used to compare the eight submaximal stages within each sub-technique. The physiological responses for TT_DP_ and TT_DS_ were compared with a paired sample *t *test, except in the case of RPE where a Wilcoxon *signed-rank* test was used. A two-way repeated-measure ANOVA (sub-technique × time-point [i.e., minutes 1–4 of the respective TT]) was used for the comparison of power output, GE, total metabolic rate, aerobic metabolic rate, anaerobic metabolic rate, and cycle characteristics. An alternative method was used for RPE, comparing sub-technique-specific grand median values with a Wilcoxon *signed-rank* test and using a Fridman test to analyze the effect of time-point based on grand median value for DP and DS per time-point. The assumption of sphericity was tested using Mauchly’s test, and for violated sphericity, the degrees of freedom were corrected using the Greenhouse–Geisser correction (i.e., epsilon ≤ 0.75). Partial eta-squared effect size (η_p_^2^) was also reported for the ANOVA tests. Bonferroni *α* corrections were applied to all ANOVA tests. Relationships between variables were assessed using linear regression analyses. For the paired *t *tests, the standardized mean difference (Hedges’ *Hg*_*av*_, effect size [ES]) was reported (calculated according to the equation provided by Lakens (2013)). For RPE, the r effect size was calculated for the Wilcoxon *signed-rank* tests, which was calculated as the *z*-value divided by the square root of *N,* and the Kendall’s W effect size was calculated for the Fridman test as the χ^*2*^-value divided by *N(K-1)* with *K* being the number of measurements per subject.

Multivariate data analysis methods were used to examine whether TT_DP_ or TT_DS_ sub-technique-specific performance (W·kg[SM]^−1^) (Y variable) could be predicted by sub-technique-specific ${\dot{\text{V}}}$O_2peak_ (ml·kg[SM]^−1^·min^−1^), anaerobic capacity (kJ·kg[SM]^−1^), and GE (%) (X variables). Prediction of TT_DP_ and TT_DS_ performance was achieved using principal component analysis and orthogonal projections to latent structures. Detailed information on these methods has been published previously (Eriksson et al. 2013) and specific application of multivariate data analysis in the prediction of performance in winter sports has also been documented (Jones et al. 2021; Nilsson et al. 2018). To evaluate the importance of specific lab test variables, for predicting TT_DP_ and TT_DS_ performance, variable influences on projection (VIP) analyses were conducted. In orthogonal projections to a latent structures model, VIP summarizes the importance of the X variables, both for the X and Y models. Within valid orthogonal projections to the latent structure's model, VIP is normalized and the average squared VIP value is 1; thus, a VIP > 1 indicates that the variable is very likely to be important for the projection, whereas values < 0.5 indicate that the variable is less likely to be important for the projection. The Statistical Package for the Social Sciences (SPSS 21, IBM Corp., Armonk, NY, USA) and SIMCA Multivariate Data Analysis Software (SIMCA 16.0, MKS AB, Umeå, Sweden) were used to carry out statistical analyses and the level of significance was set at *α* ≤ 0.05.

## Results

### Submaximal data

Statistical differences between the submaximal stages are denoted in Table 1. Significant within sub-technique main effects of speed were observed across the submaximal stages for oxygen pulse, GE, cycle rate, cycle length, absolute poling time, relative poling time, relative swing time, and poling power while DP at a 1.5° incline. Within DS at a 6.5° incline, main effects of speed across the submaximal stages were observed for oxygen pulse, cycle rate, and cycle length.

**Table 1:** Mean ± SD of speed, power output, metabolic rate, oxygen (O_2_) pulse, gross efficiency, and cycle characteristics associated with the eight submaximal stages (SUB_1-8_) of double-poling and diagonal-stride roller-skiing

	SUB_1_	SUB_2_	SUB_3_	SUB_4_	SUB_5_	SUB_6_	SUB_7_	SUB_8_
Double poling (1.5°)								
Speed (m·s^−1^)	3.60 ± 0.16	3.94 ± 0.16	4.27 ± 0.16	4.60 ± 0.16	4.94 ± 0.16	5.27 ± 0.16	5.60 ± 0.16	5.94 ± 0.16
Power output (W·kg[SM]^−1^)	1.72 ± 0.08	1.88 ± 0.08	2.04 ± 0.08	2.20 ± 0.08	2.36 ± 0.08	2.52 ± 0.08	2.68 ± 0.08	2.84 ± 0.08
Metabolic rate (W·kg[SM]^−1^)	9.9 ± 0.9	10.7 ± 0.8	11.5 ± 0.8	12.4 ± 0.8	13.3 ± 0.9	14.2 ± 1.1	15.5 ± 1.2	16.9 ± 1.2
O_2pulse_ (mL·beat^−1^·kg[BM]^−1^ × 100)^$^	25.3 ± 2.2^d−h^	25.7 ± 2.7^d−h^	26.3 ± 2. 7^d−h^	26.9 ± 3.0^e−h^	27.5 ± 3.2^ g^^,h^	28.1 ± 3.2^ g^^,h^	29.3 ± 3.2^ h^	30.7 ± 3.5
Gross efficiency (%)^#^	17.4 ± 1.6	17.7 ± 1.3	17.8 ± 1.2^ h^	17.8 ± 1.2^ h^	17.9 ± 1.2^ h^	17.8 ± 1.2^ h^	17.3 ± 1.2^ h^	16.9 ± 1.2
Cycle rate (Hz)^$^	0.69 ± 0.06^c,f^^–^^h^	0.71 ± 0.05^f−h^	0.72 ± 0.05^f–h^	0.73 ± 0.06^f–h^	0.74 ± 0.06^ g^^,h^	0.75 ± 0.05^ h^	0.78 ± 0.06^ h^	0.82 ± 0.06
Cycle length (m)^$^	5.25 ± 0.51^b−h^	5.59 ± 0.45^c−h^	5.95 ± 0.53^d−h^	6.31 ± 0.56^e−h^	6.70 ± 0.55^f−h^	7.02 ± 0.57^ h^	7.20 ± 0.61	7.32 ± 0.64
Poling time (ms)^$^	532 ± 38^b−h^	489 ± 34^c−h^	450 ± 34^d−h^	427 ± 28^e−h^	398 ± 33^f−h^	378 ± 28^ g^^,h^	358 ± 27^ h^	336 ± 26
Swing time (ms)	944 ± 105	935 ± 82	967 ± 126	976 ± 105	973 ± 106	983 ± 113	946 ± 84	932 ± 102
Relative poling time (% of cycle)^$^	36.2 ± 3.0^b−h^	34.4 ± 2.5^c−h^	31.9 ± 3.4^d−h^	30.6 ± 2.9^e−h^	29.2 ± 2.7^f−h^	27.9 ± 2.9^ h^	27.5 ± 2.1	26.6 ± 2.3
Relative swing time (% of cycle)^$^	63.8 ± 3.0^b−h^	65.6 ± 2.5^c−h^	68.1 ± 3.4^d−h^	69.4 ± 2.9^e−h^	70.8 ± 2.7^f−h^	72.1 ± 2.9^ h^	72.5 ± 2.1	73.4 ± 2.3
Poling power (W·kg[SM]^−1^)^$^	4.8 ± 0.5^b−h^	5.5 ± 0.6^c−h^	6.5 ± 0.9^d−h^	7.3 ± 0.9^e−h^	8.2 ± 0.9^f–h^	9.1 ± 1.1^ g^^,h^	9.8 ± 0.9^ h^	10.7 ± 1.0
Diagonal stride (6.5°)								
Speed (m·s^−1^)	1.71 ± 0.07	1.85 ± 0.07	1.99 ± 0.07	2.13 ± 0.07	2.27 ± 0.07	2.40 ± 0.07	2.54 ± 0.07	2.68 ± 0.07
Power output (W·kg[SM]^−1^)	2.28 ± 0.09	2.46 ± 0.09	2.65 ± 0.09	2.83 ± 0.09	3.02 ± 0.09	3.20 ± 0.09	3.39 ± 0.09	3.57 ± 0.09
Metabolic rate (W·kg[SM]^−1^)	11.3 ± 0.5	12.3 ± 0.6	13.2 ± 0.6	14.1 ± 0.6	15.1 ± 0.6	16.0 ± 0.7	16.9 ± 0.7	17.9 ± 0.9
O_2pulse_ (mL·beat^−1^·kg[BM]^−1^ × 100)^$^	27.5 ± 2.5^b−h^	28.1 ± 2.7^d−h^	28.6 ± 3.1^e−h^	29.2 ± 3.2^e−h^	29.8 ± 3.3^f−h^	30.2 ± 3.2^ g^^,h^	30.9 ± 3.0^ h^	31.5 ± 3.2
Gross efficiency (%)	20.2 ± 0.8	20.0 ± 0.7	20.1 ± 0.7	20.1 ± 0.6	20.0 ± 0.7	20.1 ± 0.7	20.0 ± 0.6	20.0 ± 0.7
Cycle rate (Hz)^$^	0.66 ± 0.18^e−h^	0.66 ± 0.18^e−h^	0.66 ± 0.18^e−h^	0.68 ± 0.18^f−h^	0.69 ± 0.19	0.69 ± 0.19	0.70 ± 0.19	0.70 ± 0.19
Cycle length (m)^$^	2.29 ± 0.63^b−h^	2.48 ± 0.69^c−h^	2.64 ± 0.73^d−h^	2.77 ± 0.76^e−h^	2.91 ± 0.79^f−h^	3.06 ± 0.83^ g^^,h^	3.19 ± 0.87^ h^	3.40 ± 0.92

^$^*P* < 0.001 for within-sub-technique main effect. ^#^*P* < 0.01 for within-sub-technique main effect. ^§^*P* ≤ 0.05 for within-sub-technique main effect

^a^Significantly different from SUB_1_ (*P* < 0.05)

^b^Significantly different from SUB_2_ (*P* < 0.05)

^c^Significantly different from SUB_3_ (*P* < 0.05)

^d^Significantly different from SUB_4_ (*P* < 0.05)

^e^Significantly different from SUB_5_ (*P* < 0.05)

^f^Significantly different from SUB_6_ (*P* < 0.05)

^*g*^Significantly different from SUB_7_ (*P* < 0.05)

^h^Significantly different from SUB_8_ (*P* < 0.05)

No statistical comparisons were performed for speed, power output, and metabolic rate

*SM* system mass, *BM* body mass, *O*_2pulse_, oxygen pulse

### 4-min TT data

**Fig. 1:**
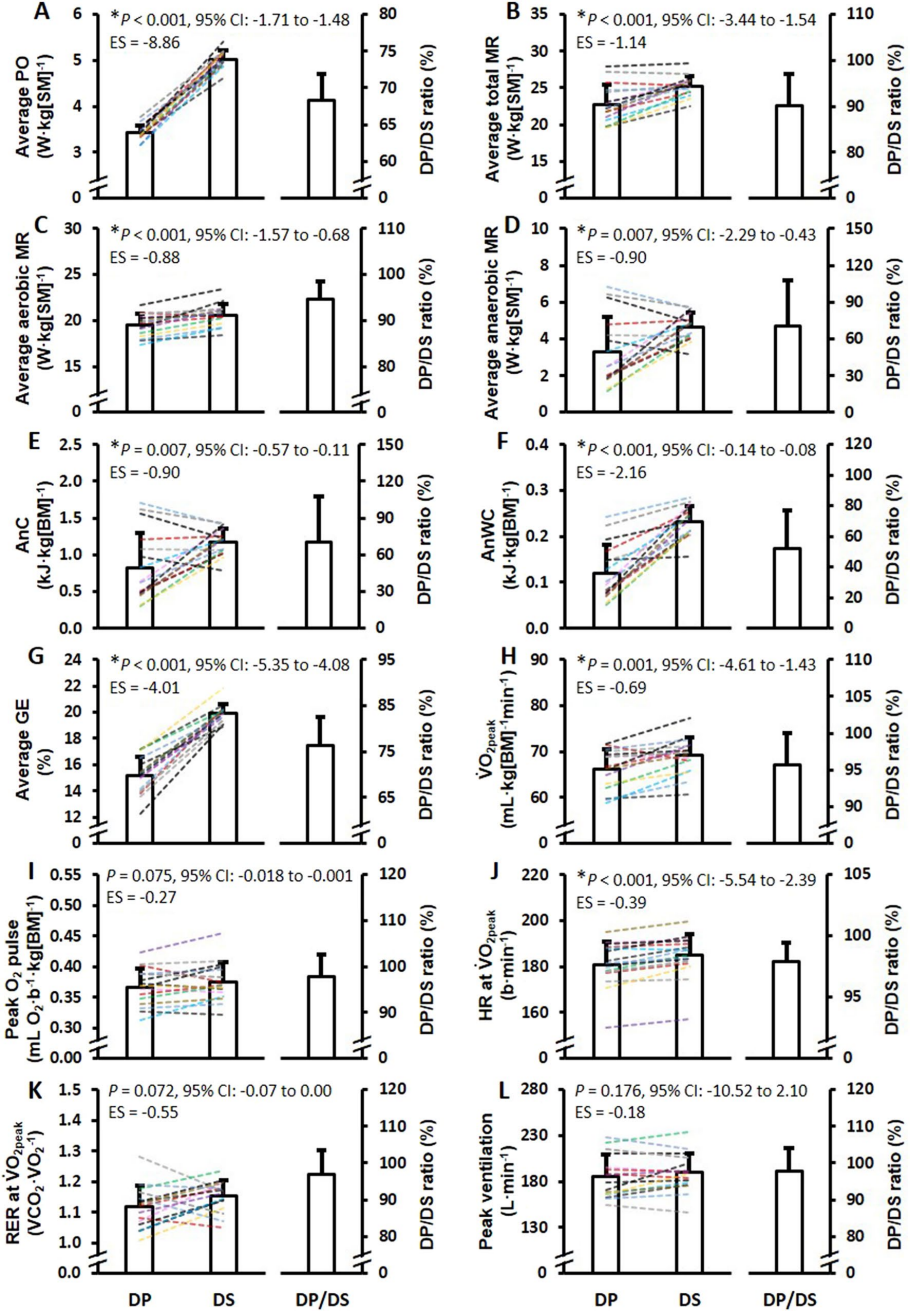
Mean ± SD power output (PO) and physiological responses to the 4-min treadmill roller-skiing time-trial tests using the double-poling (DP) and diagonal-stride (DS) sub-techniques at uphill gradients of 1.5° and 6.5°, respectively. Abbreviations: MR, metabolic rate; AnC, anaerobic capacity; AnWC, anaerobic work capacity; GE, gross efficiency; $\dot{\mathrm{V}}$O_2peak_, peak oxygen uptake; O_2_, oxygen; HR, heart rate; RER, respiratory exchange ratio; BM, body mass; SM, system mass; 95% CI, 95% confidence interval of the mean difference; ES, effect size (Hedges’ *g*_*av*_ effect size). *Significant difference between conditions (*P* < 0.05)

**Fig. 2:**
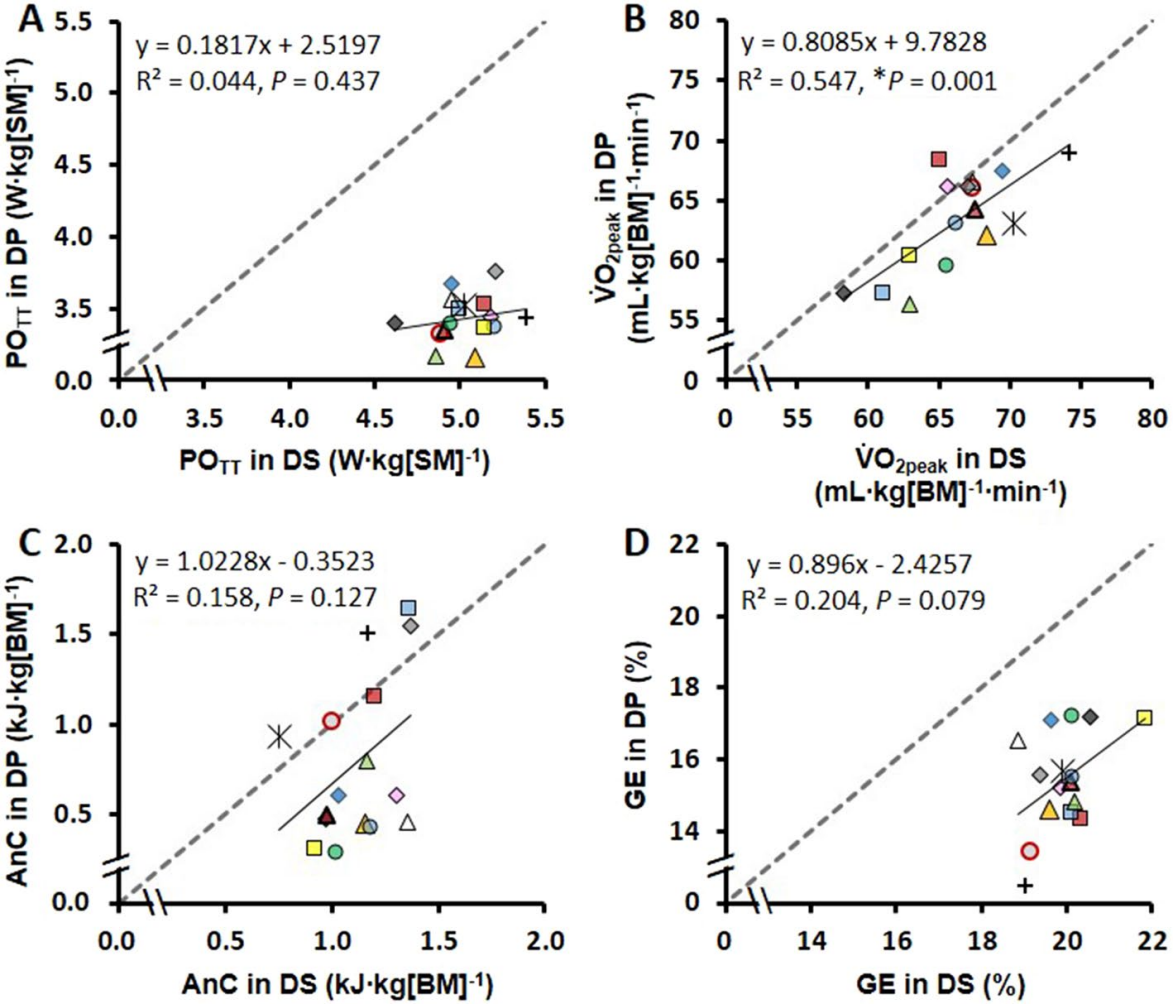
Linear relationships between performance **A** and physiological responses **B**–**D** for the 4-min diagonal-stride (DS) time-trial (TT) at 6.5° (*x*-axis) and for the 4-min double-poling (DP) TT at 1.5° (*y*-axis). Abbreviations: PO_TT_, average time-trial power output (i.e., performance); $\dot{\mathrm{V}}$O_2peak_, peak oxygen uptake; AnC, anaerobic capacity; GE, gross efficiency; SM, system mass; BM, body mass. The gray dashed line represents the identity line (i.e., *y* = *x*). *Significant *R*^2^ (*P* < 0.05)

Mean ± SD power output and physiological responses during the “flat” TT_DP_ (1.5°) and uphill TT_DS_ (6.5°) are shown in Fig. 1. Average power output, average total metabolic rate, average aerobic metabolic rate, average anaerobic metabolic rate, anaerobic capacity, anaerobic work capacity, average GE, ${\dot{\text{V}}}$O_2peak_, and heart rate at ${\dot{\text{V}}}$O_2peak_ were all significantly lower during TT_DP_ than TT_DS_ (Fig. 1A–H, J). The average ventilation rate was similar for TT_DP_ and TT_DS_ (165 ± 20 and 165 ± 18 L·min^−1^, 95% CI of the difference: –7.31 to 7.21, *P* = 0.988, ES = 0.00). The average breathing frequency was significantly higher for TT_DP_ than TT_DS_ (62 ± 5 and 54 ± 7 breaths·min^−1^, 95% CI of the difference: 3.43 to 13.20, *P* = 0.002, ES = 1.33). Both average cycle rate and cycle length were significantly higher for TT_DP_ than TT_DS_ (cycle rate: 1.04 ± 0.08 and 0.86 ± 0.04 Hz, 95% CI of the difference: 0.12 to 0.34, *P* < 0.001, ES = 1.35; cycle length: 6.94 ± 0.57 and 4.40 ± 0.16 m, 95% CI of the difference: 2.14 to 3.50, *P* < 0.001, ES = 3.03). The blood lactate concentration measured 2 min after the TT was not significantly different between TT_DP_ and TT_DS_ (11.7 ± 2.1 and 12.6 ± 2.5 mmol·L^−1^, 95% CI of the difference: –2.23 to 0.55, *P* = 0.216, ES = -–0.35). The RPE was significantly lower for TT_DP_ than TT_DS_ (9.0 [IQR: 9.0–10.0] and 10.0 [IQR: 9.8–10.0], *P* = 0.014, ES (r) = -–0.62). Between-sub-technique specific linear relationships for power output, ${\dot{\text{V}}}$O_2peak_, anaerobic capacity, and GE as based on the separate TT performances are shown in Fig. 2A–D.

### Pacing

**Fig. 3:**
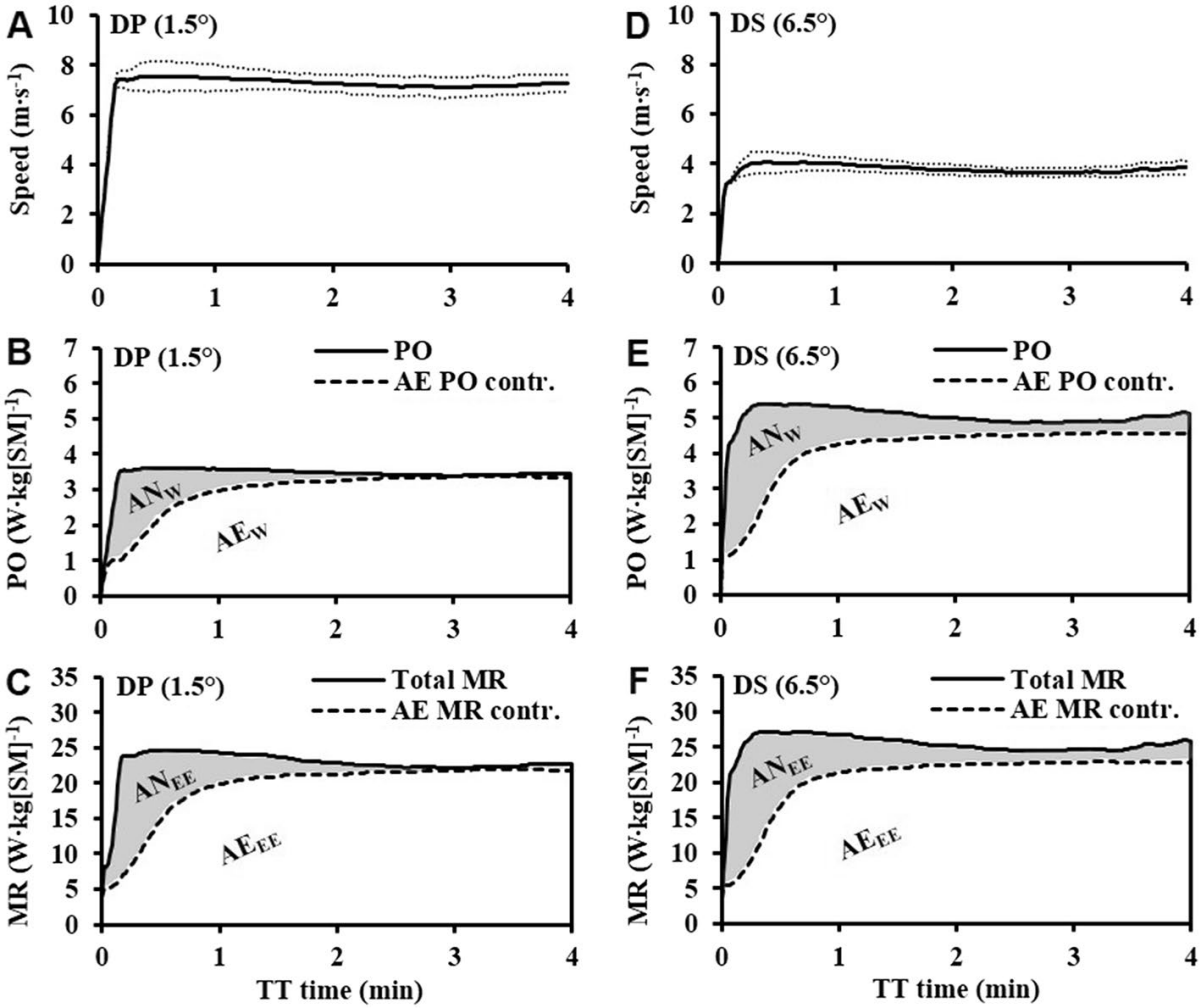
Mean ± SD speed, mean power output (PO), mean aerobic contribution to PO (AE PO contr.), mean total metabolic rate (MR), mean aerobic contribution to MR (AE MR contr.) for the 4-min double-poling (DP) time-trial (TT), in panels A–C, and for the 4-min diagonal stride (DS) TT, in panels D-F. The shaded area in panel B and E, respectively, represents the anaerobically attributable work (AN_W_) (i.e., the anaerobic work capacity), and the shaded area in panel C and F, respectively, represent the anaerobic energy expenditure (AN_EE_) (i.e., the anaerobic capacity). The white area in panels B and E, respectively, represents the aerobically attributable work (AE_W_), and the white area in panels C and F, respectively, represents the aerobic energy expenditure (AE_EE_). PO and MR are expressed relative to system mass (SM), i.e., the sum of body mass and equipment mass

Mean ± SD instantaneous speed, mean instantaneous power output, mean instantaneous aerobic contribution to power output, mean instantaneous total metabolic rate, and mean instantaneous aerobic contribution to metabolic rate for TT_DP_ and TT_DS_ are shown in Fig. 3A–F. Statistical comparisons between each of the four 1-min segments for the TT_DP_ versus TT_DS_ are presented in Table 2. For the sub-technique comparison, power output, GE, total metabolic rate, aerobic metabolic rate, anaerobic metabolic rate, and RPE were all lower during TT_DP_ than TT_DS_, whereas cycle rate and cycle length were higher during TT_DP_ (Table 2). Significant main effects of time-point were observed for power output, GE, total metabolic rate, aerobic metabolic rate, anaerobic metabolic rate, cycle rate, and RPE (Table 2). Significant sub-technique × time-point (minutes 1–4 of the TT) interactions were observed for power output, GE, and cycle length, whereas no interactions were observed for total metabolic rate, aerobic metabolic rate, and anaerobic metabolic rate.

**Table 2:** Mean ± SD for mean power output, gross efficiency, total metabolic rate, aerobic metabolic rate, anaerobic metabolic rate, cycle rate, cycle length, and rating of perceived exertion for the double-poling (DP) and diagonal-stride (DS) sub-techniques during each 1-min interval of the 4-min time-trial

	Minute	DP (1.5°)	DS (6.5°)	Effect	Test statistic	*P*	ES
Power output (W·kg^−1^[SM])	1	3.35 ± 0.21^b^	5.10 ± 0.39	Sub-technique*	F_1.0,15_ = 879.9	*P* = < 0.001	0.983
	2	3.53 ± 0.17^c^	5.14 ± 0.27^c^	Time-point†	F_1.9,28_ = 3.4	*P* = 0.049	0.185
	3	3.43 ± 0.18	4.90 ± 0.21	Interaction††	F_1.8,27_ = 4.1	*P* = 0.032	0.214
	4	3.43 ± 0.17	4.97 ± 0.25				
Gross efficiency (%)	1	14.9 ± 1.5	19.9 ± 0.7	Sub-technique*	F_1.0,15_ = 250.4	*P* = < 0.001	0.943
	2	15.1 ± 1.5^c^	19.9 ± 0.7	Time-point†	F_1.4,21_ = 6.4	*P* = 0.013	0.299
	3	15.4 ± 1.4	19.9 ± 0.7	Interaction††	F_1.4,21_ = 7.2	*P* = 0.008	0.326
	4	15.4 ± 1.3	19.9 ± 0.7				
Total metabolic rate (W·kg^−1^[SM])	1	22.7 ± 3.4	25.7 ± 2.2	Sub-technique*	F_1.0,15_ = 31.2	*P* = < 0.001	0.675
	2	23.6 ± 3.0^c^	25.9 ± 1.7^c^	Time-point†	F_1.7,26_ = 3.8	*P* = 0.040	0.204
	3	22.4 ± 2.4	24.7 ± 1.5	Interaction	F_1.5,22_ = 0.9	*P* = 0.391	0.057
	4	22.5 ± 2.2	25.0 ± 1.5				
Aerobic metabolic rate (W·kg^−1^[SM])	1	13.6 ± 1.1^b,c,d^	14.9 ± 1.1^b,c,d^	Sub-technique*	F_1.0,15_ = 29.0	*P* = < 0.001	0.659
	2	20.9 ± 1.4^c,d^	22.0 ± 1.4^c,d^	Time-point†	F_1.7,26_ = 1074.8	*P* = < 0.001	0.986
	3	21.6 ± 1.5^d^	22.7 ± 1.3	Interaction	F_1.8,26_ = 1.3	*P* = 0.279	0.081
	4	21.9 ± 1.4	22.8 ± 1.3				
Anaerobic metabolic rate (W·kg^−1^[SM])	1	9.1 ± 3.0^b,c,d^	10.7 ± 1.6^b,c,d^	Sub-technique*	F_1.0,15_ = 9.7	*P* = 0.007	0.394
	2	2.7 ± 2.2^c,d^	3.8 ± 1.0^c,d^	Time-point†	F_1.7,26_ = 286.2	*P* = < 0.001	0.950
	3	0.8 ± 1.7	2.0 ± 1.1	Interaction	F_1.4,20_ = 0.6	*P* = 0.508	0.037
	4	0.6 ± 1.6	2.1 ± 0.8				
Cycle rate (Hz)	1	1.04 ± 0.10	0.87 ± 0.04	Sub-technique*	F_1.0,14_ = 109.1	*P* = < 0.001	0.886
	2	1.06 ± 0.09	0.87 ± 0.04	Time-point†	F_1.8,25_ = 3.9	*P* = 0.037	0.218
	3	1.02 ± 0.07	0.86 ± 0.04	Interaction	F_1.8,25_ = 1.2	*P* = 0.316	0.078
	4	1.03 ± 0.09	0.84 ± 0.06				
Cycle length (m)	1	6.75 ± 0.54^b,c^	4.41 ± 0.27	Sub-technique*	F_1.0,14_ = 343.2	*P* = < 0.001	0.961
	2	7.01 ± 0.61	4.45 ± 0.24^c^	Time-point	F_1.8,26_ = 2.5	*P* = 0.108	0.150
	3	7.03 ± 0.59	4.31 ± 0.21	Interaction††	F_1.6,23_ = 3.8	*P* = 0.045	0.214
	4	6.99 ± 0.63	4.44 ± 0.25				
Rating of perceived exertion (0–10)	1	7.0 (5.8–8.0)^b,c,d^	7.0 (6.8–8.0)^b,c,d^	Sub-technique*	*Z* = −2.5	*P* = 0.011	0.633
	2	8.0 (7.0–8.3)^c,d^	9.0 (8.0–9.0)^d^	Time-point†	χ^2^ = 42.0	*P* = < 0.001	0.875
	3	9.0 (8.0–9.0)	9.0 (9.0–10.0)	–	–	–	–
	4	9.0 (9.0–10.0)	10.0 (9.8–10.0)				

*ES* effect size, *SM* system mass. ANOVA statistics are presented for sub-technique, time-point, and interaction effects, respectively, except for RPE where non-parametric statistics are presented for sub-technique and time-point. Partial eta-squared effect size is presented for all variables with exception of the rating of perceived exertion where r and Kendall's W effect sizes are presented for sub-technique and time-point effects, respectively

^b^Significantly different from minute 2 (*P* < 0.05)

^c^Significantly different from minute 3 (*P* < 0.05)

^d^Significantly different from minute 4 (*P* < 0.05)

*Significant two-way ANOVA effect for sub-technique (*P* < 0.05)

†Significant two-way ANOVA effect for time-point (*P* < 0.05). ††Significant two-way ANOVA effect for interaction (*P* < 0.05)

### Performance determinants and time-trial performance

**Fig. 4:**
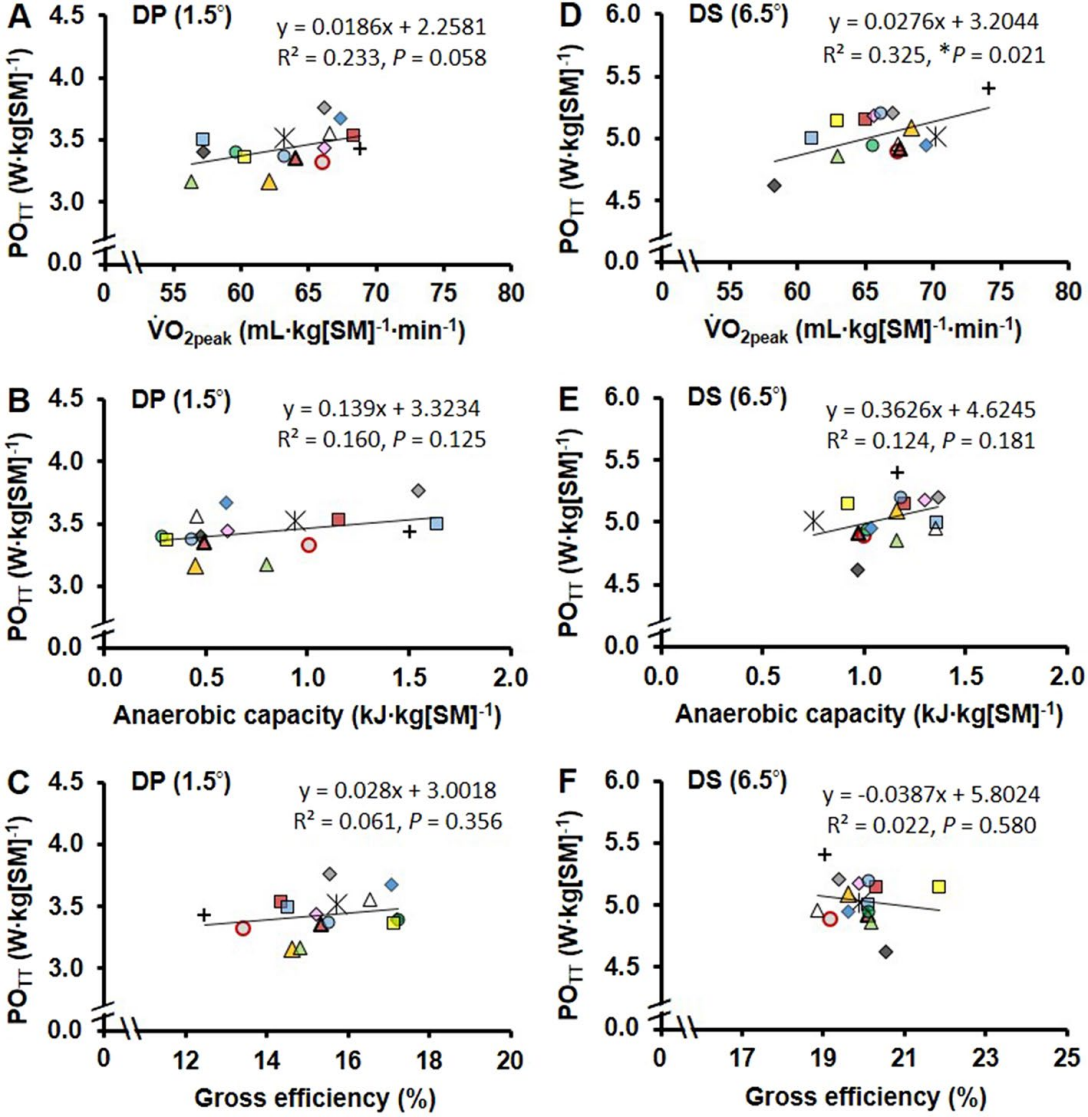
The 4-min time-trial power output (PO_TT_) in relationship with peak oxygen uptake ($\dot{\mathrm{V}}$O_2peak_), anaerobic capacity, and gross efficiency for the 4-min double-poling (DP) time-trial, in panels **A**–**C**, and for the 4-min diagonal-stride (DS) time-trial, in panels **D**–**F**. The data points represent the individual skiers together with the linear regression line. PO_TT_, $\dot{\mathrm{V}}$O_2peak_, and anaerobic capacity are expressed relative to system mass (SM), i.e., the sum of body mass and equipment mass. *Significant *R*^2^ (*P* < 0.05)

**Fig. 5:**
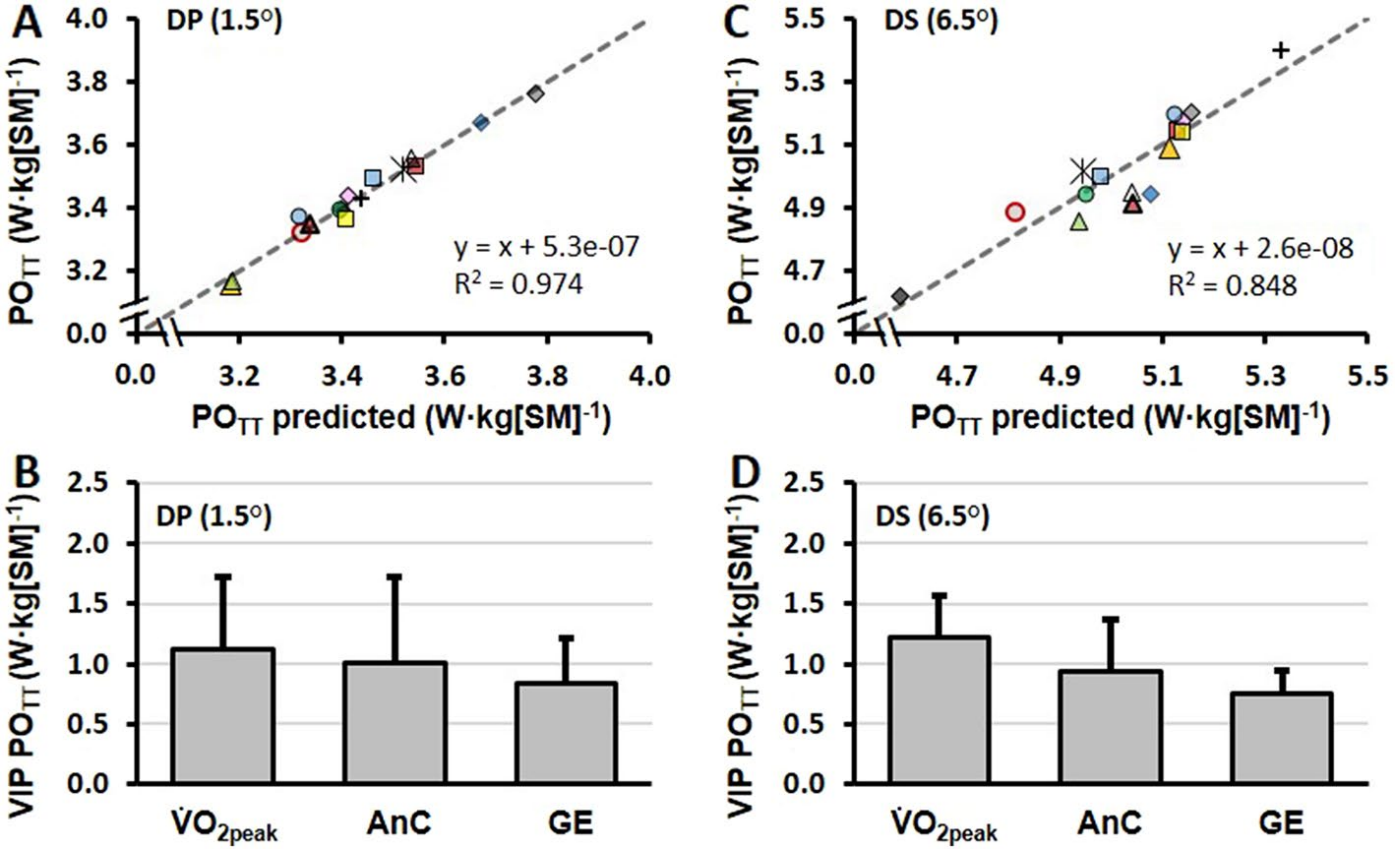
Regression coefficients of the underlying models for predicting new observations of double-poling (DP) (Panel A) and diagonal-stride (DS) (Panel C) time-trial power output (PO_TT_) including lines of best fit. The importance of peak oxygen uptake ($\dot{\mathrm{V}}$O_2peak_ [mL·kg[SM]^−1^·min^−1^]), anaerobic capacity (AnC [kJ·kg[SM]^−1^]), and gross efficiency (GE [%]), i.e., the X variables, for predicting DP (Panel B) and DS (Panel D) time-trial performances (i.e., the Y variables). Characteristics with variable influence on projection (VIP) values (in arbitrary units) where higher values indicate a higher relevance for explaining Y. The plot is displayed with 95% jackknife uncertainty bars

For each respective TT, linear relationships between ${\dot{\text{V}}}$O_2peak_ versus average power output, anaerobic capacity versus average power output, and GE versus average power output are presented in Fig. 4A–F. Valid predictive models were identified for TT_DP_ and TT_DS_ performance. The combination of variables including ${\dot{\text{V}}}$O_2peak_ (mL·kg[SM]^−1^·min^−1^), anaerobic capacity (kJ·kg[SM]^−1^), and GE (%), was able to predict TT_DP_ (R2/Q2 adjusted = 0.97/0.96) and TT_DS_ (R2/Q2 adjusted = 0.85/0.70) performance. The regression coefficients of the underlying models for predicting new observations of TT_DP_ and TT_DS_ performance are presented in Fig. 5A and C. The importance of ${\dot{\text{V}}}$O_2peak_, anaerobic capacity, and GE in predicting TT_DP_ and TT_DS_ performance are presented in Fig. 5B and D. Although all three physiological variables were of importance in the prediction of TT_DP_ and TT_DS_ performances, the most important was ${\dot{\text{V}}}$O_2peak_, which was followed by anaerobic capacity as the second most important variable, and GE as the least important variable, with the same order of importance in both TT_DP_ and TT_DS_.

## Discussion

This study compared the physiological responses and pacing strategies between a “flat” TT_DP_ (1.5° incline) and an uphill 4-min TT_DS_ (6.5° incline) with the main findings as follows: (a) compared to TT_DS_, TT_DP_ generated 32% lower power output, 10% lower total metabolic rate, 5% lower aerobic metabolic rate, and 32% lower anaerobic metabolic rate; (b) TT_DP_ resulted in 4.7 percentage points lower GE, 30% lower anaerobic capacity, and 4% lower ${\dot{\text{V}}}$O_2peak_ than TT_DS_; (c) as based on average power output (in W·kg[SM]^−1^), the TT_DP_ and TT_DS_ performances were not significantly correlated; and (d) multivariate data analysis methods were able to predict performance using ${\dot{\text{V}}}$O_2peak_, anaerobic capacity, and GE (TT_DP_, *R*^2^ = 0.974; TT_DS_, *R*^2^ = 0.848) with ${\dot{\text{V}}}$O_2peak_ being the most important variable, anaerobic capacity the second most important variable, and GE the least important variable for each respective TT projection.

The importance of the DP sub-technique has increased during the last decade and has resulted in physiological adaptations (Stöggl and Holmberg 2011; Stöggl et al. 2019, 2020). For example, over an approximately 60-year period, the ${\dot{\text{V}}}$O_2peak_ in DP relative to ${\dot{\text{V}}}$O_2peak_ in DS has increased from 70% (in the year of 1961) up to 95% (in the year of 2018) (Stöggl et al. 2019) and may be related to a combination of several factors such as changes in training characteristics, skiing technique, skiing equipment, and track preparation. In the current study, the DP-to-DS ${\dot{\text{V}}}$O_2peak_ ratio was 96%, which is similar to some recent findings (Andersson et al. 2017; Stöggl et al. 2019) but higher than the consensus finding of 88% (Losnegard 2019). The 96% of DP-to-DS ${\dot{\text{V}}}$O_2peak_ that was observed in the current study indicates that some senior-elite male cross-country skiers may have an even smaller gap in ${\dot{\text{V}}}$O_2peak_ between the two sub-techniques, possibly due to more specific upper body training. A training regime that emphasizes more specific DP training for reducing the sub-technique-specific gap in ${\dot{\text{V}}}$O_2peak_ may be advantageous, since highly trained cross-country skiers may have difficulties in increasing their ${\dot{\text{V}}}$O_2max_ (or ${\dot{\text{V}}}$O_2peak_) in DS.

The two main differences between DP and DS that were observed in the current study were the considerably lower anaerobic capacity and GE in DP, which were the main variables to explain the 32% lower power output during the 4-min TT_DP_ than TT_DS_. The lower anaerobic capacity for almost flat DP versus uphill DS roller-skiing may be due to the lower total muscle mass involved (Björklund et al. 2015) and higher muscle contraction velocities during DP (Hill 1922; Lindinger and Holmberg 2011; Lindinger et al. 2009). As an example, in running, Sloniger et al. (1997) reported lower values of anaerobic capacity for horizontal versus uphill running that in part was explained by the larger active muscle volume during uphill running. During high-speed DP (> 25 km h^−1^), the short poling times (< 300 ms) (Losnegard 2019) may limit the ability for force impulse generation and may result in a lower active muscle mass than during uphill DS. This could also explain the slightly lower RPE values for DP (post-RPE = 9) than DS (post-RPE = 10) that were observed in the current study. Moreover, Losnegard and Hallén (2014) proposed that the total active muscle mass is highly related to the magnitude of the oxygen deficit (i.e., anaerobic capacity) and the lower active muscle mass in DP than in DS, as has been observed by Björklund et al. (2015), may, at least in part, explain the sub-technique specific difference in anaerobic capacity that was observed in the current study.

Due to the substantially lower GE in DP than DS, the difference in anaerobic work capacity between DP and DS was considerably larger (43% lower in DP) than the noticed difference in anaerobic capacity (30% lower in DP). The lower GE for almost flat DP than uphill DS may be related to several factors such as the physiological characteristics and contraction velocities of the involved muscle groups (Calbet et al. 2005; Hill 1922). One main difference between almost flat DP and uphill DS is the relative propulsive phase that is considerably shorter during DP than DS (Pellegrini et al. 2011) and is a factor that may explain some of the differences in GE between the two sub-techniques. In addition, GE in DP was found to be speed dependent (see Table 1), whereas GE in DS was independent of speed. As demonstrated in Table 1, the speed-GE dependency in DP is likely to be related to the gradually shorter poling phase (both absolutely and relatively), the higher required poling power, and the more rapid increase in cycle rate for DP at higher speeds (speeds > 5.6 m·s^−1^). The GE in DP peaked during submaximal speeds ranging between approximately 4.3–5.3 m·s^−1^, with poling times, cycle rates, and poling powers likely to be the most “optimal” for GE in that speed range.

Although the DP and DS ${\dot{\text{V}}}$O_2peak_ values were linearly associated (*R*^2^ = 0.547), the TT_DP_ and TT_DS_ performances were not significantly associated (*R*^2^ = 0.044) (Fig. 2A–B). In addition, anaerobic capacity in DP was not significantly associated with anaerobic capacity in DS, and a similar finding was observed for GE (Fig. 2C–D). These results suggest that several physiological and anthropometrical factors that may favor DP performance do not necessarily favor DS performance. The locomotion of the DP and DS sub-techniques is also very different (Pellegrini et al. 2013), and for an effective DP technique, well-developed upper body strength and endurance are likely to be more crucial than in DS (Stöggl et al. 2019). Such factors may also explain why a high anaerobic capacity in DS does not directly transfer to a high anaerobic capacity in DP.

Pacing can from an internal metabolic perspective be described as the distribution of total metabolic rate (i.e., the sum of both aerobic and anaerobic metabolic rates) during a maximal effort (Andersson et al. 2016; Foster et al. 2003). In the current study, 4-min self-paced maximal efforts were performed with DP and DS, respectively. As shown in Fig. 3, the time-course profiles of speed, power output, and metabolic rates were relatively similar between DP and DS. The absence of significant sub-technique × time-point interactions for total metabolic rate, aerobic metabolic rate, and anaerobic metabolic rate confirms that similar parabolic pacing strategies were used from an internal metabolic perspective. Self-paced roller-skiing TT performance in a laboratory is related to total metabolic rate (i.e., the sum of aerobic and anaerobic metabolic rates) multiplied by GE which determines the magnitude of the total metabolic rate that is being converted to external power output (Andersson et al. 2017). As demonstrated in Fig. 4, the pair-wise linear relationships between ${\dot{\text{V}}}$O_2peak_, anaerobic capacity, and GE versus performance revealed DS ${\dot{\text{V}}}$O_2peak_ to be significantly associated with the TT_DS_ performance, whereas none of the other pair-wise linear relationships showed significance.

The multivariate data analysis method revealed that ${\dot{\text{V}}}$O_2peak_, anaerobic capacity, and GE predicted both TT_DP_ and TT_DS_ performance to a large extent (*R*^2^ = 0.974 in DP and *R*^2^ = 0.848 in DS). The VIP (i.e., variable influence on projection) values were highest for ${\dot{\text{V}}}$O_2peak_ in both TT_DP_ and TT_DS_, followed by anaerobic capacity, and GE (for details, see Fig. 5A–D). Based on these findings, all three variables had a relatively large influence on predicting performance in this group of highly trained cross-country skiers. The somewhat lower projective ability of anaerobic capacity than ${\dot{\text{V}}}$O_2peak_ for TT performance observed here may, at least in part, be related to the fact that most of the energy turnover was aerobic (on average 86% and 81% during TT_DP_ and TT_DS_, respectively). In connection with classic-style mass-start races and sprint knock-out heats where athletes race head-to-head, the relative importance of anaerobic factors (power and capacity) to race performance is likely to be even higher, because such races usually are finished with a brief high-speed end spurt involving DP. In addition to the anaerobic factors, the high variability in submaximal GE during DP is likely to play a crucial role in races. The between-athlete variability observed was approximately twice as high for DP than DS with the respective coefficient of variations of 7.1 and 3.5% at the highest submaximal speed. At high supramaximal DP speeds, the between-athlete variability in GE is likely to be even higher due to the short time for force generation (Losnegard 2019; Stöggl and Holmberg 2011). Due to this, GE may be an important performance determinant for maximal DP speed that is likely to be linked to a skier’s muscle strength, muscle power, and technical characteristics (Stöggl and Holmberg 2011).

The lack of significant pair-wise linear relationships between ${\dot{\text{V}}}$O_2peak_, anaerobic capacity, and GE versus TT performance indicate that the physiological characteristics of cross-country skiers versus their performance ability in DP and DS are very heterogeneous. This together with the fact that all the three main performance factors (i.e., ${\dot{\text{V}}}$O_2peak_, anaerobic capacity, and GE) were important in the projection of TT performance (see Fig. 5) which suggest that all three factors should be evaluated, sub-technique-specifically, during a cross-country skier’s training year. For this purpose, a roller-skiing TT is likely to be preferable compared to an incremental ${\dot{\text{V}}}$O_2max_ time-to-exhaustion test as it provides a more ecologically valid and reliable measure of performance (McGawley 2017) and can be used to determine aerobic and anaerobic metabolic responses as well as pacing strategies that all are important to performance (Andersson et al. 2016; Losnegard et al. 2012, 2013). For example, Losnegard et al. (2013) showed in the V2 ski-skating sub-technique (also referred to as G3) that movement economy and anaerobic capacity changed significantly during a 1-year training/racing period, whereas ${\dot{\text{V}}}$O_2peak_ remained constant across the year despite changes in performance. Moreover, Sandbakk et al. (2016b) showed that sub-technique-specific performance based on a 3-min roller-skiing TT in a laboratory-predicted section-specific performance during a traditional 10-km classic-style cross-country skiing race. Altogether, the results of the current study and previous findings (Losnegard et al. 2013; Sandbakk et al. 2016b) indicate that sub-technique-specific performance factors in DP and DS should be tested during the training season, so that an athlete’s strengths and weaknesses can be identified, which could allow for appropriate individual evaluation and/or adjustments in an athlete’s training.

In conclusion, the main physiological differences between TT_DP_ and TT_DS_ were related to the somewhat lower aerobic metabolic rate (5%) and substantially lower anaerobic metabolic rate (30%). The conversion of total metabolic rate to external power output was considerably lower during TT_DP_ than TT_DS_ with a considerably lower GE for TT_DP_ than TT_DS_, which resulted in a 32% lower external power output during TT_DP_ than TT_DS_. The current study reveals that highly trained male cross-country skiers can attain a ${\dot{\text{V}}}$O_2peak_ during “flat” DP which is 96% of the ${\dot{\text{V}}}$O_2peak_ attained during uphill DS but can only generate an anaerobic capacity that is 70% of the DS value. The time-course distribution of total metabolic rate during the 4-min TT did not differ between the two sub-techniques, which based on an internal metabolic perspective confirms that similar parabolic pacing strategies were used. The ${\dot{\text{V}}}$O_2peak_, anaerobic capacity, and GE predicted both TT_DP_ and TT_DS_ performances with ${\dot{\text{V}}}$O_2peak_ and anaerobic capacity having the greatest projective ability.

### Perspectives

The results of the current study demonstrate that a skier’s “metabolic profile” is sub-technique specific and that highly trained male cross-country skiers can reach a very high fraction of their “whole-body” exercise ${\dot{\text{V}}}$O_2peak_ (i.e., ${\dot{\text{V}}}$O_2peak_ in DS) during DP. The results also show that skiers that perform well in DP do not necessarily perform well in DS. The findings also show that the anaerobic capacity (or GE) in one sub-technique is not directly related to the anaerobic capacity (or GE) in the other sub-technique, which indicates that both physiological characteristics and cross-country skiing performance are highly related to the type of sub-technique being used. Even though the physiological characteristics were different for DP and DS exercise, similar parabolic pacing strategies were used during TT_DP_ and TT_DS_. Since the overall race performance in a traditional cross-country skiing race is the sum of all the sub-technique-specific performances (Sandbakk et al. 2016b), a testing procedure using a 4-min roller-skiing TT to identify key-performance-related physiological variables in each of the main sub-techniques of cross-country skiing may be a prerequisite for optimal training evaluation. It is therefore important to assess ${\dot{\text{V}}}$O_2peak_ (or ${\dot{\text{V}}}$O_2max_), anaerobic capacity, and GE, on a sub-technique-specific level, as all three variables were shown to be important in the projection of 4-min “sprint-skiing” performance.

## Data Availability

Data are available on request.

## Abbreviations

ANOVA: Analysis of variance
DP: Double poling
DS: Diagonal stride
FIS: The International Ski Federation
ES: Hedges’ *g* effect size
GE: Gross efficiency
IQR: Interquartile range
MR_AE_: Aerobic metabolic rate
MR_AN_: Anaerobic metabolic rate
MR_TT_req_: The required metabolic rate during the 4-min time-trial
PO_TT_: Power output during the time-trial
RER: Respiratory exchange ratio
RPE: Rating of perceived exertion
SD: Standard deviation
SM: System mass
TT: Time trial
TT_DP_: The 4-min double-poling time-trial
TT_DS_: The 4-min diagonal-stride time-trial
VIP: Variable influence on projection
$${\dot{\text{V}}\text{O}}_{{2}}$$: Oxygen uptake
${\dot{\text{V}}\text{O}}_{{{\text{2max}}}}$: Maximal oxygen uptake
${\dot{\text{V}}\text{O}}_{{{\text{2peak}}}}$: Peak oxygen uptake

## References

[CR1] Ainegren M, Carlsson P, Tinnsten M (2008). Rolling resistance for treadmill roller skiing. Sports Eng.

[CR2] Andersson EP, McGawley K (2018). A comparison between different methods of estimating anaerobic energy production. Front Physiol.

[CR3] Andersson E, Holmberg HC, Ørtenblad N, Björklund G (2016). Metabolic responses and pacing strategies during successive sprint skiing time trials. Med Sci Sports Exerc.

[CR4] Andersson E, Björklund G, Holmberg HC, Ørtenblad N (2017). Energy system contributions and determinants of performance in sprint cross-country skiing. Scand J Med Sci Sports.

[CR5] Andersson EP, Noordhof DA, Lögdal N (2020). The anaerobic capacity of cross-country skiers: the effect of computational method and skiing sub-technique. Front Sports Act Living.

[CR6] Andersson EP, Hämberg I, Do Nascimento Salvador PC, McGawley K (2021). Physiological responses and cycle characteristics during double-poling versus diagonal-stride roller-skiing in junior cross-country skiers. Eur J Appl Physiol.

[CR7] Bergh U, Kanstrup IL, Ekblom B (1976). Maximal oxygen uptake during exercise with various combinations of arm and leg work. J Appl Physiol.

[CR8] Björklund G, Holmberg HC, Stöggl T (2015). The effects of prior high intensity double poling on subsequent diagonal stride skiing characteristics. Springerplus.

[CR9] Calbet JA, Holmberg HC, Rosdahl H, van Hall G, Jensen-Urstad M, Saltin B (2005). Why do arms extract less oxygen than legs during exercise?. Am J Physiol Regul Integr Comp Physiol.

[CR10] Eriksson L, Byrne T, Johansson E, Trygg J, Vikström C (2013). Multi-and megavariate data analysis basic principles and applications.

[CR11] Foster C, Florhaug JA, Franklin J, Gottschall L, Hrovatin LA, Parker S, Doleshal P, Dodge C (2001). A new approach to monitoring exercise training. J Strength Cond Res.

[CR12] Foster C, De Koning JJ, Hettinga F, Lampen J, La Clair KL, Dodge C, Bobbert M, Porcari JP (2003). Pattern of energy expenditure during simulated competition. Med Sci Sports Exerc.

[CR13] Gløersen Ø, Gilgien M, Dysthe DK, Malthe-Sørenssen A, Losnegard T (2020). Oxygen demand, uptake, and deficits in elite cross-country skiers during a 15-km race. Med Sci Sports Exerc.

[CR14] Hill AV (1922). The maximum work and mechanical efficiency of human muscles, and their most economical speed. J Physiol.

[CR15] Jeukendrup A, Saris WH, Brouns F, Kester AD (1996). A new validated endurance performance test. Med Sci Sports Exerc.

[CR16] Jones TW, Lindblom HP, Karlsson Ø, Andersson EP, McGawley K (2021). Anthropometric, physiological, and performance developments in cross-country skiers. Med Sci Sports Exerc.

[CR17] Laaksonen MS, Andersson EP, Jonsson Kårström M, Lindblom H, McGawley K (2020). Laboratory-based factors predicting skiing performance in female and male biathletes. Front Sports Act Living.

[CR18] Lakens D (2013). Calculating and reporting effect sizes to facilitate cumulative science: a practical primer for t-tests and ANOVAs. Front Psychol.

[CR19] Lindinger SJ, Holmberg HC (2011). How do elite cross-country skiers adapt to different double poling frequencies at low to high speeds?. Eur J Appl Physiol.

[CR20] Lindinger SJ, Stöggl T, Müller E, Holmberg HC (2009). Control of speed during the double poling technique performed by elite cross-country skiers. Med Sci Sports Exerc.

[CR21] Losnegard T (2019). Energy system contribution during competitive cross-country skiing. Eur J Appl Physiol.

[CR22] Losnegard T, Hallén J (2014). Physiological differences between sprint- and distance-specialized cross-country skiers. Int J Sports Physiol Perform.

[CR23] Losnegard T, Myklebust H, Hallén J (2012). Anaerobic capacity as a determinant of performance in sprint skiing. Med Sci Sports Exerc.

[CR24] Losnegard T, Myklebust H, Spencer M, Hallén J (2013). Seasonal variations in VO2max, O2-cost, O2-deficit, and performance in elite cross-country skiers. J Strength Cond Res.

[CR25] McGawley K (2017). The reliability and validity of a four-minute running time-trial in assessing VO2max and performance. Front Physiol.

[CR26] McKay AKA, Stellingwerff T, Smith ES, Martin DT, Mujika I, Goosey-Tolfrey VL, Sheppard J, Burke LM (2022). Defining training and performance caliber: a participant classification framework. Int J Sports Physiol Perform.

[CR27] Nilsson R, Lindberg AS, Theos A, Ferguson RA, Malm C (2018). Aerobic variables for prediction of alpine skiing performance - a novel approach. Sports Med Int Open.

[CR28] Noakes TD (2008). Testing for maximum oxygen consumption has produced a brainless model of human exercise performance. Br J Sports Med.

[CR29] Pellegrini B, Bortolan L, Schena F (2011). Poling force analysis in diagonal stride at different grades in cross country skiers. Scand J Med Sci Sports.

[CR30] Pellegrini B, Zoppirolli C, Bortolan L, Holmberg HC, Zamparo P, Schena F (2013). Biomechanical and energetic determinants of technique selection in classical cross-country skiing. Hum Mov Sci.

[CR31] Sagelv EH, Engseth TP, Pedersen S, Pettersen SA, Mathisen G, Heitmann KA, Welde B, Thomassen TO, Stöggl TL (2018). Physiological comparisons of elite male visma ski classics and national level cross-country skiers during uphill treadmill roller skiing. Front Physiol.

[CR32] Sandbakk Ø, Hegge AM, Losnegard T, Skattebo Ø, Tønnessen E, Holmberg HC (2016). The physiological capacity of the world's highest ranked female cross-country skiers. Med Sci Sports Exerc.

[CR33] Sandbakk Ø, Losnegard T, Skattebo Ø, Hegge AM, Tønnessen E, Kocbach J (2016). Analysis of classical time-trial performance and technique-specific physiological determinants in elite female cross-country skiers. Front Physiol.

[CR34] Sloniger MA, Cureton KJ, Prior BM, Evans EM (1997). Anaerobic capacity and muscle activation during horizontal and uphill running. J Appl Physiol.

[CR35] Stöggl T, Holmberg HC (2011). Force interaction and 3D pole movement in double poling. Scand J Med Sci Sports.

[CR36] Stöggl T, Björklund G, Holmberg HC (2013). Biomechanical determinants of oxygen extraction during cross-country skiing. Scand J Med Sci Sports.

[CR37] Stöggl T, Ohtonen O, Takeda M, Miyamoto N, Snyder C, Lemmettylä T, Linnamo V, Lindinger SJ (2019). Comparison of exclusive double poling to classic techniques of cross-country skiing. Med Sci Sports Exerc.

[CR38] Stöggl TL, Hertlein M, Brunauer R, Welde B, Andersson EP, Swarén M (2020). Pacing, exercise intensity, and technique by performance level in long-distance cross-country skiing. Front Physiol.

[CR39] Watkins J, Platt S, Andersson E, McGawley K (2017). Pacing strategies and metabolic responses during 4-minute running time-trials. Int J Sports Physiol Perform.

[CR40] Weir JB (1949). New methods for calculating metabolic rate with special reference to protein metabolism. J Physiol.

